# Surgical Single Stage Treatment for Obstructive Hypertrophic Cardiomyopathy and Aortic Arch Aneurysm

**DOI:** 10.1055/s-0040-1714124

**Published:** 2020-12-23

**Authors:** Davide Margonato, Valerio Stefano Tolva, Giuseppe Vaccari, Paolo Bianchi, Renato Casana, Gianfranco Parati, Paolo Ferrazzi

**Affiliations:** 1 Department of Cardiology, Policlinico di Monza, Monza, Italy; 2 Department of Cardiology, Fondazione Policlinico San Matteo, Pavia, Italy; 3 Department of Vascular Surgery, Policlinico di Monza, Monza, Italy; 4 Department of Cardiac Surgery, Policlinico di Monza, Monza, Italy; 5 Vascular Surgery Research Laboratory, Istituto Auxologico Italiano, Milano, Italy; 6 Cardiology Unit and Department of Cardiovascular, Neural and Metabolic Science, Istituto Auxologico Italiano, S. Luca Hospital, Milano, Italy

**Keywords:** hypertrophic cardiomyopathy, aortic arch aneurysm, aortic replacement, aortic stent graft, myomectomy, myectomy

## Abstract

Coexistence of obstructive hypertrophic cardiomyopathy and severe aortic pathology is extremely rare; nonetheless, the association between these two diseases is fascinating. Here we present a unique case report of a patient with obstructive hypertrophic cardiomyopathy and aortic arch aneurysm treated by a single surgical procedure.

## Introduction

Hypertrophic cardiomyopathy (HCM) is the most common heritable cardiovascular disease, affecting 1 out of 500 people. It is characterized by asymmetric left ventricular (LV) hypertrophy in the absence of a secondary cause, and by dynamic obstruction of the LV outflow tract (LVOT) 1 Aortic dilation is a major cause of morbidity and mortality, and its association with obstructive HCM (OHCM) has recently been investigated 2 because of common signaling pathways that may play a significant role in both diseases. 3 We present a unique case report of a patient affected by OHCM and saccular aortic arch aneurysm treated by surgical septal myectomy, mitral valve plasty, and ascending aortic replacement, as well as aortic arch endoprosthesis with concomitant upper trunk rerouting during the same surgical procedure.

## Case Presentation

**Fig. 1:**
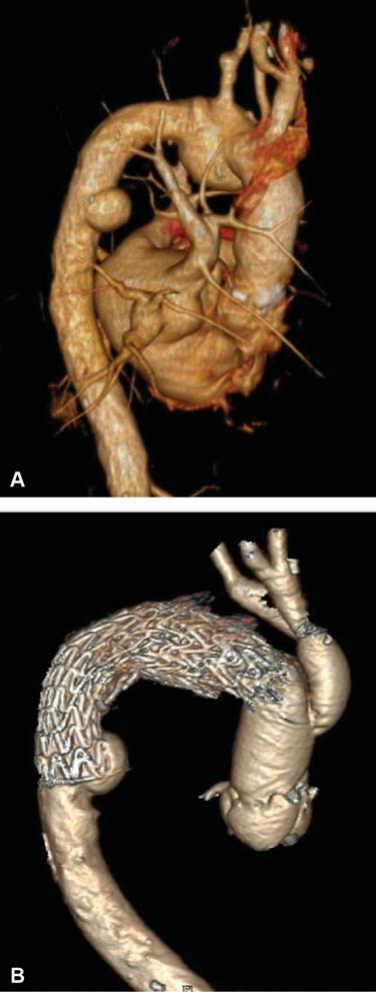
( **A** ) Presurgery computed tomography (CT) scan showing a thrombosed sacciform aortic arch aneurysm with maximum diameter of 43 mm. ( **B** ) Arch vessel vascular graft patency and complete sealing of the aortic lesion at 1-month follow-up CT scan.

A 65-year-old male with a history of OHCM was admitted to our cardiology department for elective surgical septal myectomy. OHCM was diagnosed in 2012 and treated with β blockers; however, in the last year, he developed worsening dyspnea (NYHA class III [New York Heart Association functional classification]), exercise intolerance and atrial fibrillation. Shortly before, admission chest computed tomography (CT) scan disclosed a thrombosed sacciform aortic arch aneurysm, small saccular lesion of the proximal descending aorta, and an infrarenal abdominal aortic aneurysm ( Fig. 1A ).

**Fig. 2:**
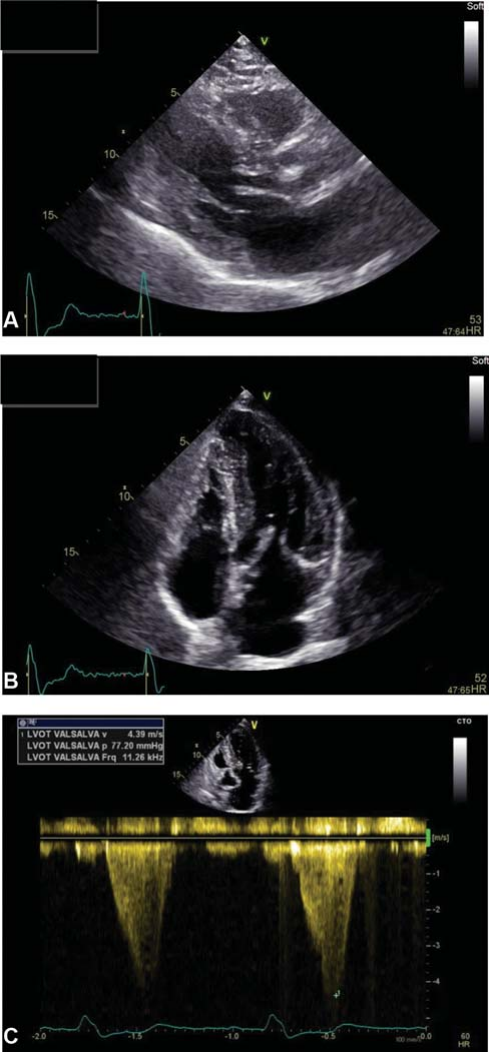
( **A** ) Preoperative transthoracic echocardiography showing anterior and ( **B** ) posterior interventricular septal hypertrophy; ( **C** ) showing significant left ventricular outflow tract obstruction after Valsalva's maneuver.

Preoperative echocardiography showed interventricular septal hypertrophy (both anterior and posterior, 19 and 17 mm, respectively, Fig. 2A and B ), systolic anterior motion of the mitral valve with LVOT obstruction (basal LVOT gradient 33 and 77 mm Hg after Valsalva's maneuver, Fig. 2C ), and normal biventricular contractility. Coronary angiography revealed no significant obstruction.

**Fig. 3:**
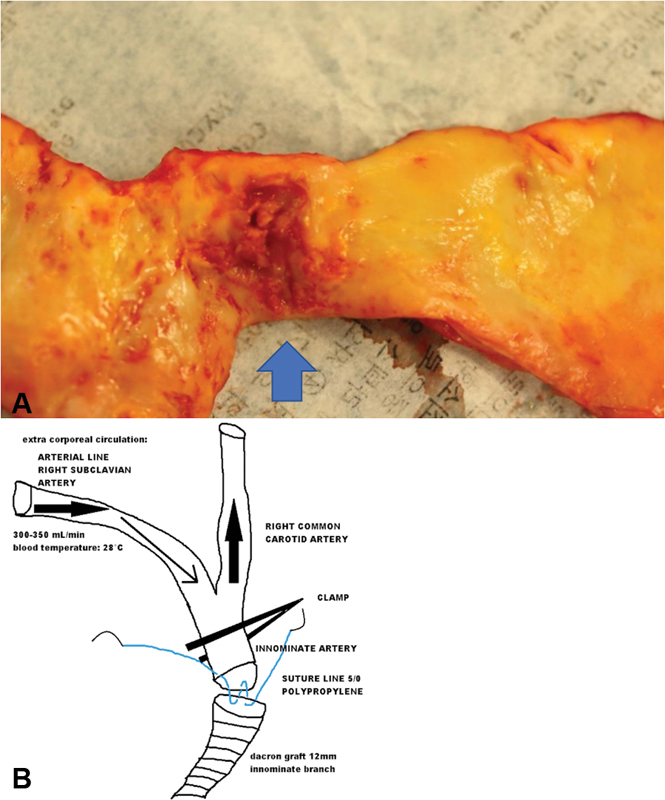
( **A** ) Intraoperative specimen reveals an ulcer-like lesion of the ascending aorta (see arrowhead). ( **B** ) Antegrade perfusion during graft and innominate trunk end-to-end anastomosis.

We decided to approach both the lesions (OHCM and aneurysm) concurrently during the same surgical procedure. We performed standard transaortic anterior septal myectomy for OHCM enriched by various interventions on the subvalvular mitral apparatus according to our experience in this technique, 4 through resection of fibrous structures connecting the papillary muscles to the ventricular septum, and resection of fibrotic secondary chordae. This intervention included ascending aorta graft repair ( Fig. 3A ; ascending aorta contained intimal ulcers), the great vessels were rerouted via an aortic bypass, and complete arch endovascular repair was performed. Ascending aorta and aortic arch were treated using a hybrid approach. A tubular 28-mm Dacron graft was used to replace the ascending aorta. A bifurcated Dacron prosthesis (24 mm × 12 mm) was sutured “end to side” to the ascending graft and “end to end” with the innominate and left common carotid artery. After aortocarotid bypass has been performed, we treated the aortic arch using a GORE C-TAG (TGMR404020E) through a right femoral artery approach. To reduce carotid clamping time and avoid cerebral ischemia, we used antegrade selective cerebral perfusion during innominate trunk anastomosis ( Fig. 3B ) using the right axillary artery as the arterial line. Ultimate angiograms showed a residual saccular aneurysm in the descending thoracic aorta that we have preoperatively decided not to treat to reduce the risk of paraplegia.

One month follow-up ( Fig. 1B ) showed vascular graft patency and complete sealing of the aortic lesion. Descending aorta aneurysm was stable in terms of morphology and diameters and we elected a regular 6-month CT scan monitoring.

## Discussion

Surgical septal myectomy is the gold-standard treatment for severe OHCM with refractory symptoms, achieving permanent resolution of the LVOT obstruction, reduction in intraventricular pressure, reduction of mitral regurgitation, and significant improvement of quality of life and long-term prognosis. 5

Recently, OHCM has been associated with aortic abnormalities. While aortic stiffness has been proven to be increased in patients with OHCM, 3 recent data have suggested an increased prevalence of aortic dilatation, although there is no clear consensus. 2 To our knowledge, this is the first case report describing a combined surgical intervention on OHCM and aortic aneurysm. Ours is a reference center for OHCM, with 570 cases surgically treated between 2013 and 2019. However, we have never before faced the coexistence of both OHCM and aortic pathology requiring contemporary surgical treatment.

Different factors could play a role in the association between aortic pathology and HCM: TGF-β (transforming growth factor-beta) overexpression, neurohormonal disturbance, endothelial dysfunction, and an abnormal baroreceptor response of the LV. 3.6 For all these reasons, an exclusive subset of patients with both OHCM and aortic aneurysm could be at higher risk for adverse events such as aneurysm rupture and dissection.

Concerning clinical decision making, whereas our patient fulfilled all the criteria of surgery for OHCM, we were not guided solely by aneurysm dimension. In this patient, a hybrid procedure instead of complete surgical arch repair was deemed to be safer for both neurological and bleeding complications.

There is no consensus regarding surgical indications for small saccular aneurysm of the aortic arch. Empirically, eccentric saccular aneurysms are thought to pose a higher risk for rupture. Although there is weak evidence, the current ACC/AHA (American College Cardiology/American Heart Association) guidelines recommend concomitant replacement of a significantly enlarged or weakened ascending aorta at the time of cardiac surgery. 7 Moreover, saccular aortic lesions are thought more prone to spontaneous rupture and standard surgical sizing criteria are considered insufficient when aortic bulging or penetrating aortic ulcer are evaluated. Therefore, the dynamic, unpredictable and global clinical picture of our patient led us to the decision of a complete approach to both the cardiac and aortic disease.
